# Cannabidiol for neurodegenerative disorders: A comprehensive review

**DOI:** 10.3389/fphar.2022.989717

**Published:** 2022-10-25

**Authors:** Sukanya Bhunia, Nagesh Kolishetti, Adriana Yndart Arias, Arti Vashist, Madhavan Nair

**Affiliations:** ^1^ Department of Immunology and Nanomedicine, Herbert Wertheim College of Medicine, Florida International University, Miami, FL, United States; ^2^ Institute of Neuroimmune Pharmacology, Herbert Wertheim College of Medicine, Florida International University, Miami, FL, United States

**Keywords:** cannabidiol, neuroinflammation, oxidative stress, neurodegenerative diseases, Alzheimer’s disease, Parkinson’s disease

## Abstract

Despite the significant advances in neurology, the cure for neurodegenerative conditions remains a formidable task to date. Among various factors arising from the complex etiology of neurodegenerative diseases, neuroinflammation and oxidative stress play a major role in pathogenesis. To this end, some phytocannabinoids isolated from *Cannabis sativa* (widely known as marijuana) have attracted significant attention as potential neurotherapeutics. The profound effect of ∆^9^-tetrahydrocannabinol (THC), the major psychoactive component of cannabis, has led to the discovery of the endocannabinoid system as a molecular target in the central nervous system (CNS). Cannabidiol (CBD), the major non-psychoactive component of cannabis, has recently emerged as a potential prototype for neuroprotective drug development due to its antioxidant and anti-inflammatory properties and its well-tolerated pharmacological behavior. This review briefly discusses the role of inflammation and oxidative stress in neurodegeneration and demonstrates the neuroprotective effect of cannabidiol, highlighting its general mechanism of action and disease-specific pathways in Parkinson’s disease (PD) and Alzheimer’s disease (AD). Furthermore, we have summarized the preclinical and clinical findings on the therapeutic promise of CBD in PD and AD, shed light on the importance of determining its therapeutic window, and provide insights into identifying promising new research directions.

## Introduction

With the increase in life expectancy worldwide, the prevalence of neurodegenerative diseases (NDs) has become a concern due to their socio-economic impact. NDs are characterized by progressive loss of structure and function of nerve cells in the central and/or peripheral nervous systems (Katsnelson et al., 2016). The structural loss of neurons and synapses including demyelination and/or neuronal death causes gradual loss of motor and/or cognitive skills, eventually leading to functional loss. Aging is found to be a major risk for NDs. Alzheimer’s disease (AD) and Parkinson’s disease (PD) are the most common NDs, while the other forms include amyotrophic lateral sclerosis (ALS), Huntington’s disease, spinal muscular atrophy, motor neuron diseases, prion diseases, and spinocerebellar ataxia. However, irrespective of their differences in pathophysiology, which are determined by observing the aggregated characteristic protein *via* neuropathological evaluation at autopsy (Dugger and Dickson 2017), chronic neuroinflammation is the fundamental pathological mechanism driving both onsets and the progression of the NDs (Kinney et al., 2018).

Oxidative stress (OS) arises from redox imbalance in the cell when the cellular antioxidant systems fail to counter the excess of reactive species. Excess accumulation of reactive species causes structural alteration in protein, DNA, and cell membrane which leads to neuronal death. Persistent inflammation also plays a major role in neurodegeneration (Glass et al., 2010). The insoluble protein aggregates and/or oxidized lipid molecules cause microglial activation, a key factor in the pathogenesis of NDs. Activated microglia release inflammatory mediators that again activate astrocytes to produce reactive species and inflammatory cytokines, which in turn further activate microglia, and thereby a feed-forward loop is established favoring chronic oxidative stress and persistent neuroinflammation. Neuronal cells, especially those present in the CNS, are more susceptible to oxidative stress due to their high metabolic rate (Salim, 2017). In aging with poor self-renewal capacity of neuronal cells and a gradual decline in activity of the antioxidant enzymes to counterbalance the excessive reactive species, neuronal death becomes prevalent (Hou et al., 2019). In addition, in aging with a retarded rate of autophagy, clearance of dysfunctional mitochondria or aggregated protein becomes difficult, further favoring the environment for chronic oxidative stress and neuroinflammation. Though the gold standards for treating such diseases, for example, l-DOPA for Parkinson’s disease and small molecule acetylcholinesterase inhibitors for AD, provide symptomatic relief (Rizek et al., 2016; Marucci et al., 2021), the discovery of druggable targets and drugs that can mitigate disease progression still remains an unmet challenge.

**FIGURE 1:**
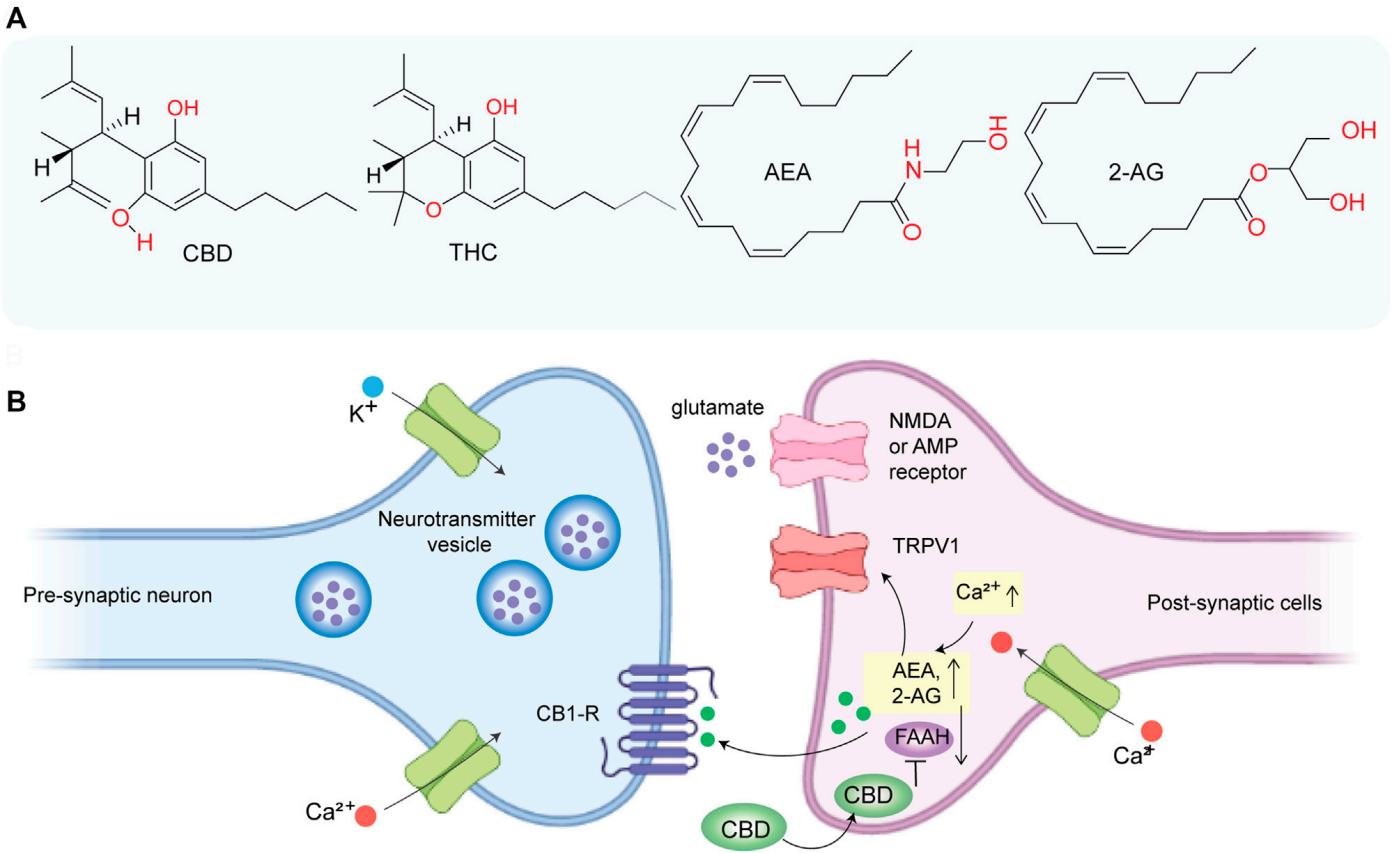
**(A)** Chemical structure of CBD, THC, anandamide (AEA), and 2-arachidonoylglycerol (2AG). **(B)** CBD indirectly activates CB1 receptor and TRPV1 by inhibiting FAAH and thereby promoting the accumulation of AEA at synapse which may play a role in inhibiting glutamate-based excitotoxicity.

For the past few decades, cannabinoids, secondary plant metabolites extracted from *Cannabis sativa*, especially ∆^9^-tetrahydrocannabinol (THC) and cannabidiol (CBD), have garnered significant attention (Cooray et al., 2020). Despite the anecdotal history of cannabis’s medicinal use since the medieval era, systematic medicinal exploration started in 1970 after its widespread use as a “recreational drug.” The profound behavioral effect of cannabis including euphoria and analgesia led to investigating the potential receptors in humans and rodents, leading to the discovery of the endocannabinoid system (ECS), which regulates several physiological processes such as cognition and analgesia. ECS consists of two primary receptors of the G-protein-coupled receptor (GPR) family: cannabinoid receptors 1 and 2 (CB1R and CB2R), two primary ligands known as endocannabinoids (namely, anandamide (AEA) and 2-arachidonoylglycerol (2AG)) and enzymes (namely, fatty acid amide hydrolase (FAAH) and monoglyceride lipase (MAGL)) that control the release and degradation of endocannabinoids (Alexander, 2016). CB1R is predominantly found in the nerve terminals of the CNS and in peripheral neural and other tissue (e.g., gastrointestinal tract) with lower density. The two endocannabinoids, AEA and 2AG (Figure 1A), act as retrograde synaptic messengers. In response to a transient intracellular Ca^2+^ elevation, endocannabinoids are released from post synapses in the synaptic space, travel to the presynaptic cleft, and activate the CB1 receptor (CB1R). Activation of CB1R blocks the release of neurotransmitters such as GABA (an inhibitory neurotransmitter) and glutamate (an excitatory neurotransmitter). AEA and 2AG are finally degraded by FAAH and MAGL, respectively. On the other hand, CB2R is mostly found in immune-associated cells and acts as an immunomodulator that can inhibit immune cell migration.

Some cannabinoids can mimic the effect of endocannabinoids and can regulate nerve impulses in certain pathways (Piomelli, 2003). For instance, THC acts as a partial agonist of the CB1 receptor (Ki = ∼10 nM) and exerts its psychoactive effect by activating the CB1 receptor (Kimura et al., 2019). Previously, major research on ECS has focused on THC, the major psychoactive component of W extract. However, recently, CBD has attracted major attention due to its neuroprotective activity (Cassano et al., 2020), its non-psychoactive nature, and well-tolerated pharmacological behavior (Bergamaschi et al., 2011). Although CBD has a low affinity for CB1 (Ki = ∼2,000 nM) (Kimura et al., 2019), it indirectly activates CB1 by inhibiting FAAH which aids in the accumulation of AEA (Figure 1B) and prevents glutamate-induced excitotoxicity on neurons (Peres et al., 2018). CBD acts as an inverse agonist of CB2R. Importantly, much of the recent evidence suggested that CBD has high anti-inflammatory and antioxidant activities which play a major role in its neuroprotective behavior, and it is mediated *via* multiple molecular targets other than CB1R and CB2R. In this review article, we have briefly discussed the role of oxidative stress and neuroinflammation in neurodegeneration and provided an overview of the general mechanism of neuroprotective behavior of CBD along with molecular targets. In addition, we have summarized clinical and preclinical findings on the therapeutic promise of CBD in PD and AD.

## Neurodegeneration: inflammation and oxidative stress

Persistent neuroinflammation and chronic oxidative stress play a crucial role in the pathogenesis of neurodegenerative diseases. Microglia cells, the immune cells of the brain, act as a “double-edged sword” (Hickman et al., 2018). It normally acts as basic housekeeping, which maintains regular neuronal function, tissue repair, and brain homeostasis. However, in the presence of persistent inflammatory stimulus, microglia shift to their activated forms, which release inflammatory mediators and reactive species that can directly cause neuronal death and activate astrocytes and neurons to express more inflammatory mediators and RS. Once the feed-forward loops of inflammatory responses are established, the normal resolution mechanisms are overwhelmed, leading to neurodegeneration (Glass et al., 2010).

The inflammatory response represents a highly regulated cascade of events containing three major types of agents: 1) inducers and sensors, 2) transduction systems, and 3) amplifiers and effectors (Glass et al., 2010). In the initiation phase, inflammation stimuli are recognized by some specific cell surface receptors of microglia. For instance, pathogen-associated molecular patterns from an infectious agent (e.g., lipopolysaccharide (LPS) from Gram-negative bacteria) are recognized by pattern recognition receptors (PRRs) as “stranger” signals, while components released from necrotic cells are recognized as “danger signals.” ATP released following traumatic injury and neuronal death is sensed by purinergic receptors on microglia and astrocytes, while the “scavenger receptors” respond to oxidized lipids, proteins, and apoptotic cells. In the second phase, activation of such cell surface-sensing receptors leads to the activation of some signal transduction pathways (e.g., IkB kinase and mitogen-activated protein kinase, i.e., MAPK), which further control the activities of multiple transcription factors such as nuclear factor-κB (NF-κB) and activator protein 1 (AP-1). These transcription factors work together to regulate the expression of cytokines (e.g., tumor necrosis factor-α (TNF-α) and interleukin-1β (IL-1β)) which propagate the inflammation (amplifier) and chemokines (e.g., COX-2) that recruit additional immune cells (effector cells) depending on the target cell. In addition, reactive species (e.g., ROS and inducible nitric oxide synthase (iNOS)) are produced as an anti-microbial defense mechanism of cells (Glass et al., 2010).

**FIGURE 2:**
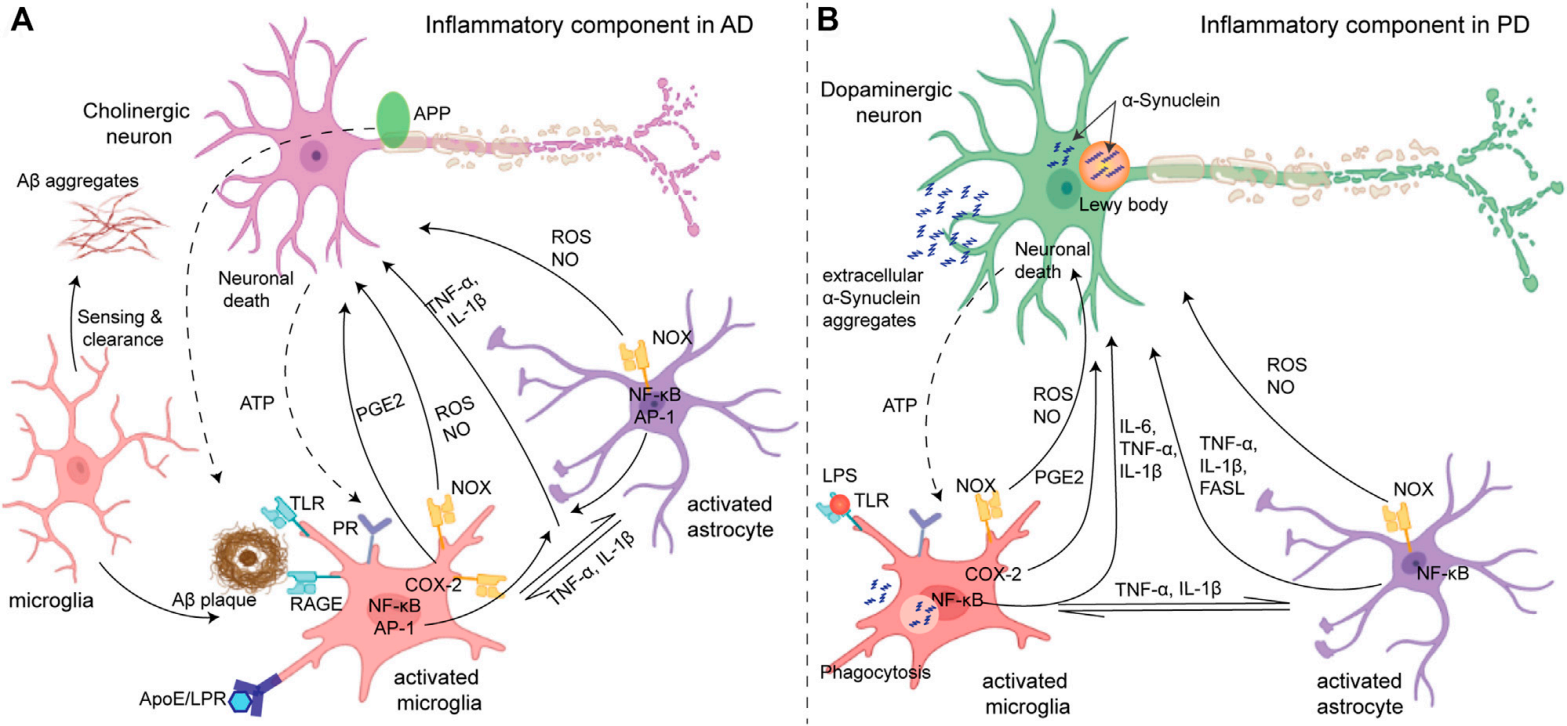
**(A)** Inflammatory components in Alzheimer’s disease (left): initially, microglia act as neuroprotectants that can sense and migrate to the Aβ aggregates. The Aβ aggregates interact with different cell surface receptors (TLR and RAGE) of microglia which induce activation of some transcription factors such as NF-kB and AP-1 and produce reactive species (ROS and NO) and cytokines (microglial activation). However, under sustained inflammation, activated microglia exhibit compromised Aβ clearance ability due to impaired ApoE/LRP uptake, while the production of ROS and inflammatory mediators remains unaltered which directly causes apoptosis/necrosis in neurons. Activated microglia can also activate astrocytes by releasing cytokines which in return can further activate the microglia. In addition, ATP released from neuronal death activates microglia *via* the purinergic receptors (PR), and collectively, a feed-forward loop is established. **(B)** Parkinson’s disease (right): intracellular deposition of α-synuclein known as Lewy bodies caused stress in dopaminergic neurons, while the oligomeric α-synuclein in the extracellular space causes activation of glia cells (microglia and astrocytes) in a similar manner to amplify the neuroinflammation and oxidative stress causing the death of dopaminergic neurons. Adapted with permission from (Glass et al., 2010).

Amyloid-β (Aβ) aggregates and neurofibrillary tangles of tau protein (NFT), the proteins involved in AD, create a stress response in neurons. These aggregates also interact with microglia and astrocytes *via* cell surface receptors (such as TLRs and the receptor for advanced glycation endproducts (RAGE)) to produce several factors, such as pro-inflammatory cytokines (e.g., TNF-α, IL-1β, and IL-6), chemokines (e.g., COX-2), ROS, NO, prostaglandins (e.g., PGE2), and caspase that promote the death of cholinergic neurons (Figure 2A). Genetic, environmental, and age-related factors contribute to the initiation of inflammation (Migliore and Coppedè, 2009; Glass et al., 2010). For instance, the expression of ApoE4, the strongest genetic factor of AD (Liu et al., 2013), causes defects in the microglial clearance of Aβ. ApoE is a lipid transporter protein that participates in the clearance of Aβ by microglia *via* a low-density lipoprotein receptor (LDL-R) or LDL-R-related protein 1 (LRP1) receptor, and the binding of ApoE to LDL-R or LRP1 suppresses expression of the stress-responsive kinase JNK pathway. However, the efficiency of ApoE in clearing Aβ is isoform-specific. The ApoE4/Aβ complex is the least preferred compared with the other isoforms of ApoE for microglial uptake by the LRP1 receptor, which results in the accumulation of Aβ with the high expression of ApoE4 (Pocivavsek et al., 2009). Other than genetic factors, environmental factors such as obesity due to a high-calorie diet also induce a low-grade but persistent inflammation. Activated microglia release TNF-α and IL-1β that activate astrocytes, which in turn activate microglia (Liu et al., 2020). Furthermore, the cholinergic neuron itself has NF-κB sites in the promoters of amyloid precursor protein (APP), presenilin, and BACE1 (beta-secretase 1) (Sastre et al., 2008) which produces more Aβ upon inflammatory stimuli and further amplifies microglia-mediated inflammation. Thus, neurons and glia act in combination to amplify the production of neurotoxic factors in AD pathology. Similarly, in PD, the process of aggregation of α-synuclein, the major factor involved in PD, from monomers to fibrils *via* oligomeric intermediate is considered to be neurotoxic, causing neuronal death (Danzer et al., 2007). Neuronal death itself and subsequent extracellularly released aggregated α-synuclein can induce microglial activation *via* the purinergic receptor (Figure 2B). Aggregated α-synuclein is sensed and internalized by microglia *via* cell surface gangliosides (Park et al., 2009), resulting in microglial activation which produces pro-inflammatory cytokines (e.g., TNF-α and IL-1β) and mediators of oxidative stress including ROS and NO (Reynolds et al., 2008). Dopaminergic neurons in the substantia nigra are vulnerable to oxidative stress (Kastner et al., 1992) due to their enhanced intracellular oxidative processes. ROS production *via* the activation of NADPH oxidase upon internalization of α-synuclein is a crucial factor for microglia activation in PD pathology.

OS plays a major role in neurodegeneration, especially in the CNS, which is vulnerable to OS due to its high metabolic rate and limited cell renewal capacity. In CNS inflammation, NADPH oxidases (NOX), NOS, and dysfunctional mitochondria are major sources of reactive species (Fischer and Maier, 2015). ROS can directly damage neurons, facilitate protein aggregates *via* impaired proteosomal degradation, and initiate inflammation *via* lipid peroxidation which causes microglial activation. In aging with a reduced rate of autophagy, the removal of dysfunctional mitochondria and aggregated proteins becomes compromised, contributing to the risk of age-related neurodegeneration. In AD, oxidative stress activates some kinases (e.g., JNK, p38 MAPK, and GSK-3β) which promote the generation of Aβ (Tamagno et al., 2005) and NFT (Lovell et al., 2004). Sustained activation of kinases also causes neuronal apoptosis. In PD, α-synuclein can directly impair the mitochondrial complex I in dopaminergic neurons which are vulnerable to oxidative stress due to their high rate of internal dopamine metabolism (Devi et al., 2008). Thus, prevention of oxidative stress and neuroinflammation may hold promise in mitigating the progression of neurodegenerative disease.

## Therapeutic benefits of CBD and general mechanism of neuroprotection

Phytocannabinoids have garnered significant attention lately due to their inherent neuroactive and strong antioxidant properties. Approval of Sativex, a combination of THC and CBD (1:1), in treating spasticity and/or neuropathic pain in multiple sclerosis (MS) in 2005, became a breakthrough in cannabis research. CBD, beyond reducing the adverse effect of THC when used in combination (Russo and Guy 2006), can individually exhibit a broad spectrum of therapeutic benefits. CBD alone has been FDA-approved for treating treatment-resistant pediatric epilepsy in 2018 (Devinsky et al., 2016). In addition, its anticonvulsant, antiemetic, and sleep-inducing properties have been explored to treat epilepsy and sleep disorders, as well as for treating psychiatric disorders such as schizophrenia, anxiety, and depression (Fernández-Ruiz et al., 2013). CBD showed a better safety profile and tolerability in patients (1,500 mg/day) than THC (Bergamaschi et al., 2011). Unlike THC, CBD did not alter cardiovascular parameters, psychomotor, and psychological functions, presumably because CBD does not directly target the CB1 receptor (Pertwee, 2006).

Emerging preclinical research studies demonstrate that CBD can exhibit neuroprotective effects due to its antioxidant and anti-inflammatory properties, which may potentially treat different neurodegenerative diseases (Hampson et al., 1998; Esposito et al., 2006; Esposito, Scuderi et al., 2007). For instance, CBD can act as a scavenger of reactive oxygen species (ROS) and inhibit lipid peroxidation, which inhibits caspase-mediated apoptosis of neurons (Iuvone et al., 2004). Interestingly, the neuroprotective effect of CBD is stronger than that of common antioxidants such as α-tocopherol or ascorbate (Atalay et al., 2019), which indicates that CBD may interact with some molecular targets. CBD has a low affinity for CB1 and CB2 receptors; however, it can indirectly activate CB1R *via* inhibition of FAAH and thereby promote the accumulation of endocannabinoids, which may prevent glutamate-mediated neurotoxicity (Pertwee 2008). In addition, CBD can also prevent glutamate-induced neurotoxicity occurring *via* the N-methyl-d-aspartate (NMDA) receptor, 2-amino-3-hydroxy-5-methyl-4-isoxazolepropionic acid (AMPA), or kainate receptor in the cannabinoid receptor-independent pathway as demonstrated by Hampson et al. (1998).

**FIGURE 3:**
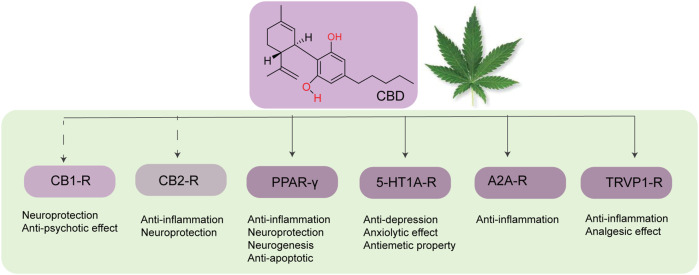
Different molecular targets of CBD in the brain.

CBD can also prevent neuroinflammation by acting on multiple molecular targets (Figure 3). CBD can reduce the expression of iNOS in neurons *via* the downregulation of NF-κB and phosphorylated p38 MAPK (Esposito et al., 2006). CBD, being an inverse agonist of the CB2 receptor (Thomas et al., 2007), can inhibit microglial activation in a CB2 receptor-associated pathway (Ramírez et al., 2005). Peroxisome proliferation-activated receptor gamma (PPARγ) (Hegde et al., 2015; O'Sullivan, 2016) is an important target of CBD. PPARγ is a member of the ligand-activated transcriptional factor of the nuclear hormone receptor superfamily, which is a key mediator of energy homeostasis and associated with many biological functions including inflammation (Daynes and Jones, 2002). Several studies have reported that PPAR-γ agonists exhibit neuroprotective effects *via* regulating gene transcription related to the pathogenesis of neurodegeneration (Kumar et al., 2020). CBD also exhibits neuroprotective and anti-inflammatory effects *via* the A_2A_ receptor where it can stimulate A_2A_-mediated signaling in immune cells by inhibiting the uptake of adenosine (Carrier Erica et al., 2006). Activation of the A_2A_ receptor by CBD decreases microglial activation in an ATP-dependent pathway (Martín-Moreno et al., 2011) and can improve cognitive and motor function *via* inhibition of TNF-α expression and increasing expression of brain-derived neurotrophic factor (BDNF) (Magen et al., 2009). In addition, CBD can also target the TRPV1 receptor to exhibit its neuroprotective effect (Giuliano et al., 2021).

CBD also has a significant effect on improving mitochondrial dysfunction. Emerging evidence suggests that mitochondrial dysfunction *via* both mitochondrial iron accumulation and ROS production is directly related to neurodegenerative disorders. To this end, da Silva et al. have demonstrated that CBD can reverse iron-induced mitochondrial dysfunction by rescuing mitochondrial ferritin and epigenetic modulation of mitochondrial DNA (da Silva et al., 2018). CBD can also act as a modest affinity agonist toward the 5-HT1A (serotonin) receptor (Russo et al., 2005), which is present in the synaptic membrane of several regions of the brain (Hoyer et al., 1986) and exhibits its anti-depression property.

## Disease-specific pathway

### CBD on Parkinson’s disease

PD is the second most prevalent neurodegenerative disease (after AD) associated with motor deficits such as tremors at rest, rigidity, and postural instability. Progressive loss of dopaminergic neurons in the midbrain substantia nigra is the pathological hallmark of PD, which results from the intracellular accumulation of Lewy bodies containing misfolded α-synuclein protein. The genetic studies reveal that the mutations in α-synuclein, PARK7, PINK1, ubiquitin genes, and population-specific gene mutations such as the glucocerebrosidase gene in Ashkenazi Jews are directly related to the onset of PD. Although the current standard of care including treatment with l-DOPA and deep brain stimulation can alleviate the symptoms, there is no therapeutic solution to date to cease the progression of neurodegeneration in PD. To this end, mitochondrial dysfunction and oxidative stress are presumed to play key roles in the pathogenesis of PD, and recent studies indicate that antioxidant phytocannabinoids such as CBD can exhibit potential therapeutic benefits due to their neuroprotective effect (Table 1). CBD is also demonstrated to improve the antioxidant defense of striatal neurons projected toward the substantia nigra (Sagredo et al., 2007). Using a rat striatum lesion, Sagredo et al. have demonstrated that CBD can completely rescue the endogenous antioxidant defense diminished by treatment with 3-nitropropionic acid, an inhibitor of mitochondrial complex II. Particularly, CBD can enhance mRNA levels for SOD-II and partially for SOD-I. Using agonists of CB1, CB2, TRPV_1_, and A2A, authors have shown that this neuroprotective effect is mediated *via* the TRPV_1_ receptor. Similarly, using a 1-methyl-4-phenylpyridinium (MPP(+))-induced cellular model of PD in SH-SY5Y and PC12 cells, Santos et al. have reported that CBD can enhance neuronal cell viability, differentiation, and the expression of synaptic (synaptophysin and synapsin I) and axonal (growth-associated protein-43) proteins (Santos et al., 2015). This neuritogenic effect of CBD against MPP(+)-induced PD is independent of nerve growth factor (NGF) but associated with the involvement of trkA receptors. A similar observation has also been reported by Gugliandolo et al. using MPP(+)-induced PD in SH-SY5Y cells (Gugliandolo et al., 2020). In this study, the authors have demonstrated that in addition to the trkA receptors, presumably ERK and AKT/mTOR pathways are also involved in the neuroprotection, which can be correlated with the interaction of CBD molecules with CB2 and TRVP1 receptors.

**TABLE 1:** CBD on PD, *in vitro*, *in vivo*, and clinical study.

Model of PD	Disease model	CBD dose and way of administration	Frequency of dosing	Major findings	Ref.
SH-SY5Y and PC12 cells	MPP(+)-induced PD	—	—	Enhanced cell viability, differentiation, and the expression of synaptic and axonal proteins	Santos et al. (2015)
SH-SY5Y cells	MPP(+)-induced PD	—	—	Neuroprotective effect *via* activation of ERK and AKT/mTOR pathways	Gugliandolo et al. (2020)
				CB2 and TRVP1 receptors as the molecular target of CBD	
Male Sprague–Dawley rats (>8 weeks)	8 μg of 6-HDA injected in the medial forebrain	3 mg/KgBW, i.p	Daily, for 2 weeks	Rescue the dopamine contents and a tyrosine hydroxylase activity in the substantia nigra	Lastres-Becker et al. (2005)
Male Sprague–Dawley rats	20 μg of 6-HDA injected in the right striatum	10 mg/kg, i.p	Daily, for 4 weeks	Improved motor performance *via* activation of astrocytic transient receptor potential vanilloid 1 (TRPV1) and enhanced expression of the endogenous neuroprotective response of ciliary neurotrophic factor (CNTF)	Giuliano et al. (2021)
PD patients (n = 7) without comorbid psychiatric conditions	—	300 mg/day, oral	Daily, for 6 weeks	Improved quality of life	Chagas et al. (2014)
PD patients (n = 4)	—	75 mg/day, oral	Daily, for 6 weeks	Improved REM sleep behavior disorder (RBD)	Chagas et al. (2014)
Six PD patients (four men and two women)	—	150 mg/day, oral	Daily, for 4 weeks	Relieved psychotic symptoms in PD patients without any adverse side effects	Zuardi et al. (2009)

The neuroprotective effect of CBD has been investigated in animal models of PD. Using a 6-hydroxydopamine (6-HDA) neurotoxin-induced PD model of rats, Ruiz and co-workers have demonstrated that a daily dose of 3 mg/KgBW of CBD over the course of 2 weeks can rescue the dopamine contents and tyrosine hydroxylase activity in the substantia nigra (Lastres-Becker et al., 2005). Interestingly, this effect is even more than that observed for the same dose of THC and is independent of the CB1 receptor. It is worth mentioning that the MPP(+)-induced animal model of PD is related to PD induced by neurotoxins, while 6-HDA-induced PD mimics the oxidative stress model of PD. To gain mechanistic insight, the authors have examined the direct effect of CBD on neuronal cultures, and neuronal cultures conditioned with media from CBD-treated glial cell culture. Authors have observed that neuroprotection is more pronounced with the conditioned media, indicating a neuroprotective role of CBD-treated glial cells. To further investigate if it is the involvement of CB receptors or the antioxidant properties of phytocannabinoids that play a key role in the observed neuroprotective behavior, PD-bearing rats were treated with small molecule inhibitors of the endocannabinoid system with and without antioxidant properties, namely, AM404 and UCM707, respectively (García-Arencibia et al., 2007). Recovery of dopamine depletion is observed when rats were treated with AM404 but not with UCM707, indicating antioxidant properties of phytocannabinoid molecules/analogue behind the neuroprotective role against PD which is further confirmed by upregulation of superoxide dismutase, a key endogenous enzyme against oxidative stress. It is worth mentioning that a recent study by the same group using a similar 6-HDA-induced cellular and murine model of PD demonstrates that the quinone derivative of CBD exhibits its neuroprotective behavior *via* PPAR-γ receptors (Burgaz et al., 2021). Furthermore, recently, Giuliano et al. have demonstrated that a higher dose of CBD (10 mg/KgBW) can reduce nigrostriatal degeneration and improve motor function in rats bearing 6-HDA-induced PD (Giuliano et al., 2021). Authors have reported that this effect is mediated *via* the activation of TRPV1 in astrocytes which further enhances expression of the endogenous neuroprotective response of ciliary neurotrophic factor (CNTF).

The effect of CBD on PD has been investigated in clinical settings too. For instance, in an exploratory double-blind clinical trial with PD patients (n = 7) without dementia or comorbid psychiatric conditions, CBD (300 mg/day) does not improve motor function significantly but has been found to improve the quality of life among the PD patients (Chagas et al., 2014). In addition, CBD also significantly improves REM sleep behavior disorder (RBD) in PD patients (n = 4) without side effects (Chagas et al., 2014). Importantly, in another open-label pilot study in six PD patients (four men and two women), CBD was found to relieve psychotic symptoms in PD patients without any adverse side effects or negative effects on motor neurons (Zuardi et al., 2009). Taken together, CBD is well tolerated in humans and effective in relieving some PD-related symptoms, indicating its potential as a complementary treatment for PD. In-depth preclinical and clinical studies with a large cohort of patients, however, are needed to further confirm this possibility.

### CBD on Alzheimer’s disease

**FIGURE 4:**
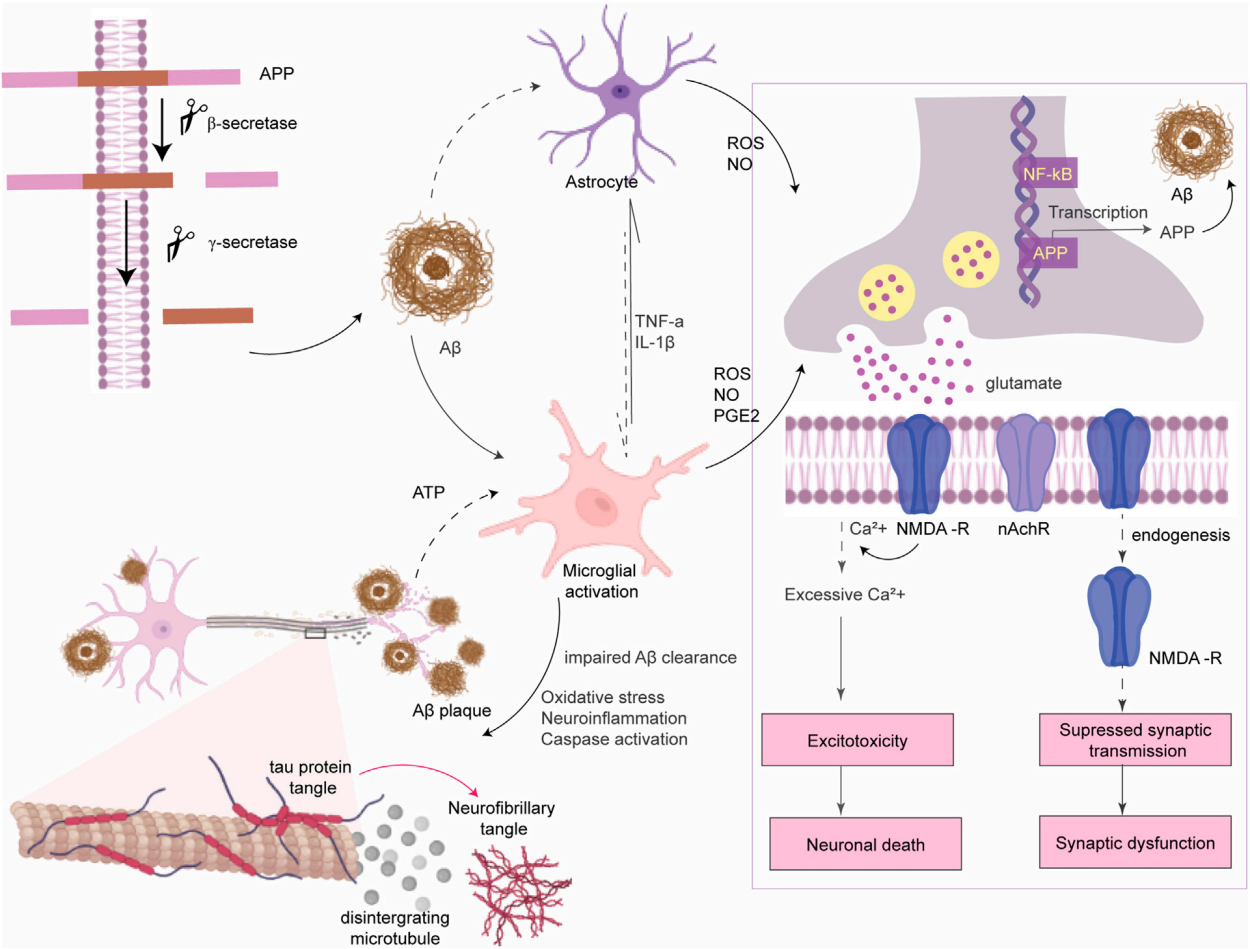
Amyloid β pathogenesis of AD. APP protein is processed by secretase enzymes to produce Aβ peptides. Aggregated Aβ causes microglial activation to produce ROS and inflammatory cytokines which further activate astrocytes. ROS binds to NF-κB sites in the promoters of APP in neurons to activate transcription of the Aβ peptide. Aβ plaques enhance glutamate release from neurons and astrocytes. Prolonged activation of extrasynaptic NMDA receptors by excess glutamate leads to intense transient Ca^2+^ influx which alters some signaling pathways and causes neuronal death (excitotoxicity). Aβ also inhibits the activity of acetylcholine esterase and lowers the expression of nicotinic acetylcholine receptor (nAchR). In addition, Aβ accumulation promotes endocytosis of NMDA receptors at synapses which suppresses synaptic transmission and alters synaptic plasticity.

AD is the most prevalent neurodegenerative disease which is characterized by progressive cognitive decline and memory dysfunction prior to the normal aging process, affecting ∼40 million people worldwide (Tiwari et al., 2019). Progression of AD is associated with a gradual loss of cholinergic neurons in the hippocampus and neocortex of the brain. The pathological hallmarks for both late-onset sporadic AD (>95% of cases) and early-onset familial AD (<5% cases) are deposition of extracellular Aβ-senile plaques, intracellular NFT of hyperphosphorylated tau protein, and loss of cholinergic neurons. Familial AD results from a genetic mutation in the APP gene or in the presenilin 1 and 2 (*PS1* and *PS2*) genes that lead to an altered ratio of amyloid β_42_ to amyloid β_40_ and eventually rapid aggregation of Aβ peptide to form Aβ plaque (Bettens et al., 2013). The cause of sporadic AD is still unclear but it is majorly influenced by environmental and lifestyle factors, with the APOE4 gene being the primary genetic risk factor (Kamboh, 2004). Irrespective of the origin of AD, Aβ accumulation initiates a cascade of events mainly including excitotoxicity, neuroinflammation, and oxidative stress that ultimately led to neurodegeneration and cognitive impairment. Aβ accumulation enhances glutamate release from astrocytes (Talantova et al., 2013) and neuronal cells (de Paula Faria et al., 2022). Excess glutamate stimulates the extrasynaptic N-methyl-d-aspartate (NMDA) receptors and causes an intense influx of Ca^2+^, which results in excitotoxicity, mitochondrial dysfunction, and neuronal cell death (Hardingham and Bading, 2010). In addition, Aβ accumulation suppresses synaptic transmission *via* promoting endocytosis of NMDA receptors at synapses (Hardingham and Bading, 2010) and alters synaptic plasticity (Figure 4) (He et al., 2019). Microglia initially clear Aβ and have neuroprotective effects. Upon persistent microglial activation, it facilitates Aβ aggregation and neurodegeneration (Cai et al., 2014).

Cholinergic neurons are majorly affected in AD with a severely diminished transcription for acetylcholine esterase enzyme (ChAT) transcription, which is closely associated with cognitive decline in AD patients (Wilcock et al., 1982). The FDA-approved drugs (three acetylcholinesterase inhibitors such as donepezil, rivastigmine, and galantamine and one NMDA receptor antagonist, namely, memantine) till date mostly target to improve the cognitive deficit *via* facilitating cholinergic neurotransmission or *via* mitigating the excitotoxicity by antagonizing the NMDA receptor. However, these therapies offer only temporary symptomatic benefits (Husna Ibrahim et al., 2020). To this end, considering the crucial role of oxidative stress and neuroinflammation in AD progression, antioxidants and nonsteroidal anti-inflammatory drugs have attracted significant attention in exploring their potential benefit to mitigate Aβ-initiated events of AD progression. In this context, cannabinoids such as CBD have garnered tremendous attention.

Since the observance of the neuroprotective effect of CBD (Hampson et al., 1998) in 1998, Esposito et al. first (in 2004) reported that pre-exposure to CBD can protect rat neuronal cells PC12 from Aβ peptide-induced neurotoxicity (Iuvone et al., 2004). CBD acts as a scavenger of ROS and dose-dependently inhibits lipid peroxidation followed by inhibition of caspase 3-mediated apoptosis of neuronal cells. To further investigate the molecular pathway of neuroprotection, the same group reported that CBD can inhibit the phosphorylation of GSK-3β (Esposito et al., 2006). GSK-3β, a kinase enzyme, plays a significant role in the pathogenesis of AD (Lauretti et al., 2020). It promotes the formation of Aβ plaque by altering the PS1/γ-secretase complex during APP cleavage and hyper phosphorylates tau promoting neurofibrillary tangle formation. It is hyperactive in the brain of AD patients and can act as a therapeutic target (Ly et al., 2013). Importantly, inhibition of GSK-3β stabilizes β-catenin, a protein present at both pre- and postsynaptic terminals, and influences synaptic size and strength (Maguschak and Ressler, 2008), which improves learning and memory deficits (Onishi et al., 2011). To this end, Esposito et al. have reported that the reduction of the phosphorylation of GSK-3β by CBD inhibits neurofibrillary tangle formation and rescue of the Wnt/β-catenin pathway (Esposito et al., 2006).

In addition to the antioxidant effect of CBD in AD, the same group has reported that CBD can also exhibit anti-inflammatory effects. iNOS and its enzymatic product NO are the major neurotoxic effectors of AD found both in Aβ-stimulated neuronal cells (Iuvone et al., 2004) and in post-mortem AD brains (Haas et al., 2002). NF-κB, a redox-sensitive transcription factor that is activated by a family of stress-activated kinases including p38 MAP kinase, regulates the expression of genes involved in oxidative, inflammatory, and immune responses. Esposito et al. have found that CBD can inhibit both NO production and iNOS protein expression at a low concentration (10^–6^ to 10^–4^ M) and significantly prevent NF-κB activation and phosphorylation of p38 MAPK in PC12 cells against Aβ insult (Esposito et al., 2006). To further investigate such anti-inflammatory effects of CBD in an animal model, the same group has also reported that early administration of CBD (i.p., 2.5 or 10 mg kg^−1^) for 7 days in C57 mice inoculated with Aβ-peptide in the hippocampus can markedly mitigate reactive gliosis by suppressing the expression of IL-1β and iNOS (Esposito et al., 2007).

Interestingly, while there is a quest for the molecular mechanism of CBD’s anti-inflammatory and neuroprotective action in AD, recent studies indicate that PPARγ plays a crucial role. An elevated expression level of PPARγ has been found in AD brain tissues (Kitamura et al., 1999). However, PPARγ agonists have been found to induce beneficial neuroprotective and anti-inflammatory effects in cultured cortical neurons (Gray et al., 2012) and improve learning and memory deficits in transgenic mice models of AD (Escribano et al., 2010). To this end, O'Sullivan et al. have reported that being a small molecule, CBD can transport to the nucleus and bind to activate the transcriptional activity of PPARγ (O'Sullivan, Sun et al., 2009). Using a rat model of AD injected with fibrillar Aβ peptide at the hippocampus, Esposito et al. have reported that CBD can mitigate Aβ-induced neuroinflammation and promote neurogenesis *via* activation of PPARγ which is diminished when CBD is used in combination with PPARγ antagonists. To further investigate the molecular pathway, authors have demonstrated that activating PPARγ by CBD diminishes Aβ-induced inflammation in cultured astrocytes *via* inhibition of NF-κB (Kozela et al., 2010; Esposito et al., 2011) and promotes ubiquitination of APP to reduce expression of Aβ peptide in neuronal SHSY5Y^APP+^ cells (Scuderi et al., 2014).

**FIGURE 5:**
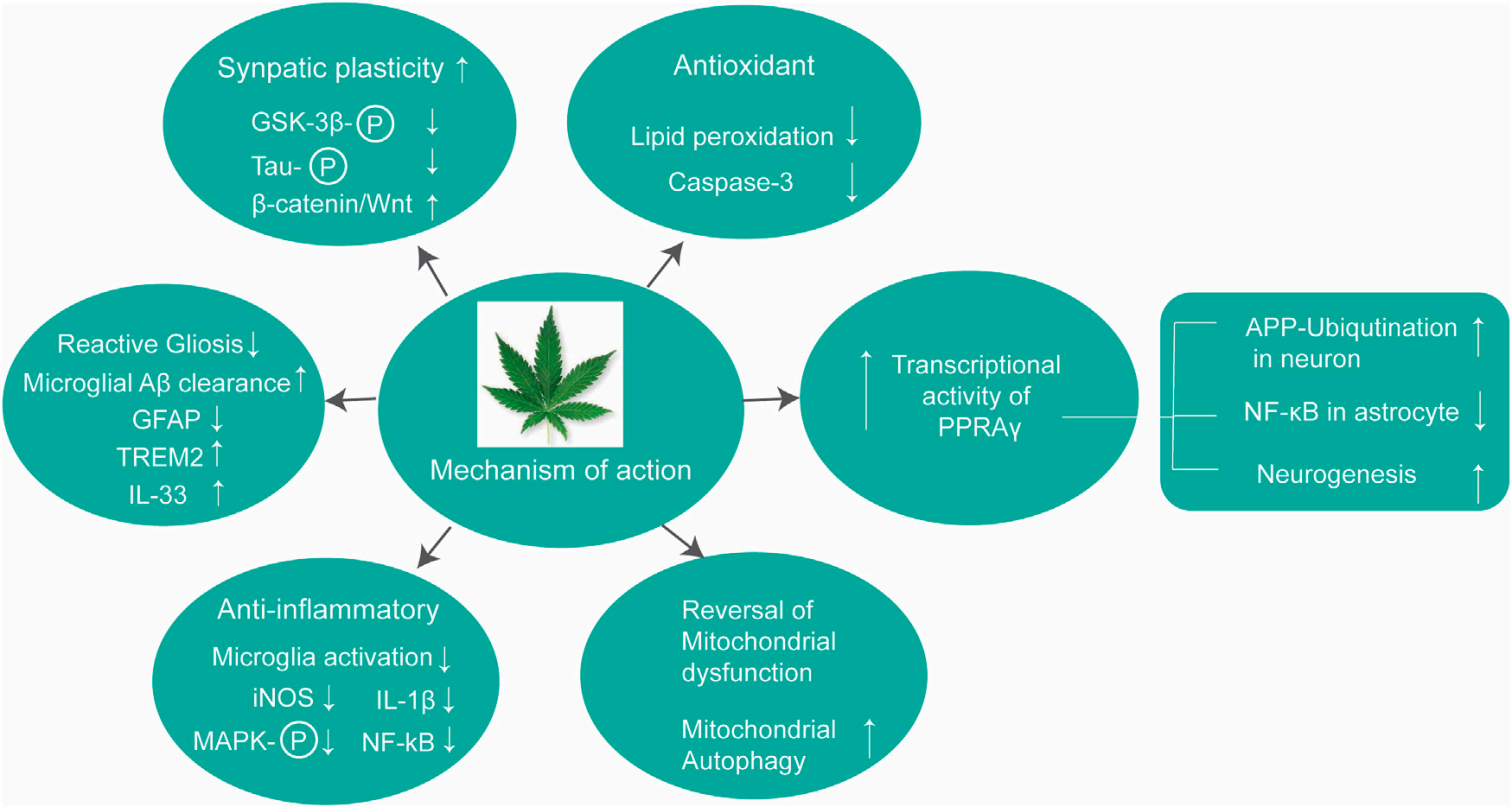
Molecular pathways of contributing neuroprotective role of CBD.

CBD can also influence other cells including microglia and mesenchymal stem cell toward neuroprotection. For instance, CBD can inhibit microglia activation in response to Aβ insult in cultured N13 microglial cells and in primary microglia cells (BV-2) presumably *via* cannabinoid and adenosine A_2A_ receptors (Martín-Moreno et al., 2011). Libro et al. have examined the regenerative potential of CBD in conditioning human mesenchymal stem cells (hMSCs) toward recovering from the neurodegeneration of AD (Libro et al., 2016). Using a transcriptomic analysis with the next-generation sequencing (NGS) study, authors have demonstrated that hMSCs pre-treated with CBD show downregulation of kinase genes responsible for tau phosphorylation, specifically the PI3K/Akt/GSK-3β pathway and secretase genes responsible for Aβ production. Using small molecule antagonists, authors have demonstrated that this effect of CDB is exhibited *via* the TRVP1 receptor and is independent of the cannabinoid receptor. CBD is also reported to enhance the migration of adipose-derived MSCs in a dose- and time-dependent manner, and this migration of MSCs is mediated *via* the CB2 receptor and GPR55 receptor by activation of the p42/44 MAPK pathway (Schmuhl et al., 2014). Cannabidiol (CBD) can act as a preventive agent to protect synaptic plasticity against AD (Hughes and Herron, 2019). Using hippocampal slices of C57BL/6J mice perfused with oligomeric beta-amyloid peptide (Aβ_1–42_) in the presence or absence of CBD treatment, Huges et al. have reported that pretreatment with CBD (not post-treatment) can recover the synaptic transmission attenuated by Aβ. The *in vitro* findings on CBD’s neuroprotective properties are summarized in Figure 5 and Table 2.

**TABLE 2:** CBD on AD, *in vitro* study.

Cell used	Major effects on CBD treatment	Ref.
PC12 cells	Protects PC12 neuronal cells from Aβ peptide (1 ug/mL)-induced neurotoxicity upon pre-exposure *via* inhibition of lipid peroxidation and downregulation of caspase 3	Iuvone et al. (2004)
PC12 cells	Inhibits Aβ-induced hyperphosphorylation of tau protein *via* reduction of the phosphorylation of GSK-3β	Esposito et al. (2006)
PC12 cells	Inhibits production of NO and iNOS protein *via* the downregulation of NF-κB and phosphorylated p38 MAPK	Esposito et al. (2006)
Culture astrocytes and primary rat astrocytes	PPARγ-mediated inhibition of reactive gliosis (suppresses GPAF expression) and neuroinflammation	Esposito et al. (2011)
Bv-2 microglia cells	Inhibition of LPS-induced microglial activation by the downregulation of NF-κB and IFN (beta)-STAT pathways	Kozela et al. (2010)
Neuronal SHSY5Y^APP+^ cells	Promotes ubiquitination of APP and lowers the expression of Aβ peptide by activating PPARγ	Scuderi et al. (2014)
Cultured N13 microglial cells and primary microglia cells (BV-2)	Reduces microglial activation *via* CB2 and A2A receptor-mediated inhibition of ATP-induced increase in intracellular Ca^2+^ and thereby microglial cell migration in addition to the anti-inflammatory effect of CBD.	Martín-Moreno et al. (2011)
Human mesenchymal stem cells (hMSCs)	Pretreatment downregulates kinase responsible for tau phosphorylation, specifically the PI3K/Akt/GSK-3β pathway and secretase genes responsible for Aβ production. Effect is mediated *via* TRVP1 receptor	Libro et al. (2016)
Mesenchymal stem cells	Migration of MSc is mediated *via* the CB2 receptor and the GPR55 receptor by activation of the p42/44 MAPK pathway	Schmuhl et al. (2014)
Hippocampal slice of C57 mice perfused with Aβ	Pretreatment recovers the synaptic transmission attenuated by Aβ	Hughes and Herron (2019)

The neuroprotective efficacy of CBD has also been evaluated using different animal models of AD including pharmacological rodent models of partial AD in C57BL/6J and Wistar rats where the disease is induced by injecting Aβ peptide on hippocampus and in established transgenic mice (Table 3). Cheng et al. have first evaluated the efficacy of CBD in a double transgenic mouse model of familial AD (*APPxPS1* mice) containing mutations in both the APP and PS1 genes. Notably, such mice start developing Aβ plaques as early as 4–6 months of age which further increases with age and exhibits a sexual dimorphism profile with female *APPxPS1* mice showing higher pathological levels of AD markers compared to males. Authors have demonstrated that daily treatment of CBD (20 mg/kg) for 3 weeks rescued social recognition memory and object recognition deficits in 6-month-old male mice with no effect on fear-associated memory or anxiety measures (Cheng et al., 2014). A similar behavioral outcome has also been observed when *APPxPS1* mice are treated in prophylactic settings with the same dose of CBD for 8 months (Cheng et al., 2014). However, 20 mg/kg CBD does not alter amyloid load or oxidative damage, although it impacts neuroinflammation and cholesterol. A recent study demonstrates that 50 mg/kg CBD can moderately reduce Aβ load in the hippocampus of a 12-month-old *APPxPS1* male along with restoration of social recognition memory and spatial learning deficit (Watt et al., 2020). In contrast, some other studies reported that small to medium doses of CBD, for example, at a dose of 0.75 mg/kg of CBD (Aso et al., 2015) or 5 mg/kg of CBD (Coles et al., 2020) can improve the cognitive deficit in *APPxPS1* mice. Some cannabinoids exhibit biphasic dose responses (Rey et al., 2012); therefore, it is crucial to investigate a range of dosages to estimate the window of the therapeutic effectiveness of the drug.

**TABLE 3:** CBD on AD, *in vivo* study.

Animal model of AD	AD pathogenesis	CBD dose and way of administration	Frequency of dosing	Major findings	Ref.
3–5-month-old C57BL/6J mice	Implantation of 10 ng of Aβ (1–42) into the right dorsal hippocampus	2.5 or 10 mg kg^−1^, i.p	Daily, 7 consecutive days, dosing started at day 3 post inoculation	Suppression of neuroinflammation *via* reducing glial fibrillary acidic protein (GFAP) mRNA, iNOS, and IL-1β protein expression	Esposito et al. (2007)
Adult male Sprague–Dawley rats (300–350 g)	Intrahippocampal injection of 30 ng fibrillar Aβ (1–42) peptide	i.p., 10 mg/kg	Daily, 15 consecutive days	Restoration of hippocampal neurogenesis *via* activating the PPARγ axis	Esposito et al. (2011)
C57/Bl6 mice, 3 months old	Intraventricular injection of 2.5 μg of fibrillar Aβ	i.p., 20 mg/kg	Daily for first 7 days, alternate days following 15 days, treatment started at Day 1	Promotes microglial cell migration, prevention of Aβ-induced cognitive deficit as determined by in the Morris water maze behavioral study	Martín-Moreno et al. (2011)
APPxPS1 mice*,* 6 months old male	Genetically modified	i.p., 20 mg/kg	Daily, 3 weeks	Rescue of social recognition and object recognition deficits	Cheng et al. (2014)
APPxPS1 mice*,* 2.5 months old	Genetically modified	Oral, 20 mg/kg	8 months	Rescue of social recognition and object recognition deficits	Cheng and Spiro et al. (2014)
APPxPS1 mice*,* 12 months old	Genetically modified	i.p., 50 mg/kg	Daily, 3 weeks	On social recognition memory and spatial learning deficits, moderate brain region-specific reductions in insoluble Aβ40 levels	Watt et al. (2020)
APPxPS1 mice	Genetically modified	CBD-rich cannabis extract (at a dose of 0.75 mg/kg of CBD)	—	Preservation of memory, reduced astrogliosis, microgliosis, and inflammatory-related molecules in treated mice, THC + CBD exhibits better than individual	Aso et al. (2015)
12-month-old *APPxPS1* female mice	Genetically modified	i.p., 5 mg/kg CBD	Daily, 3 weeks	Reverses object recognition memory deficits	Coles et al. (2020)
5xFAD mice	Genetically modified	—	—	Interleukin (IL)-33 and triggering receptor expressed on myeloid cell 2 (TREM2) which reduces cognitive decline	Khodadadi et al. (2021)
Male 6-month-old APP/PS1 mice	Genetically modified	i.p. with 5 mg/kg BW of CBD	Daily for 30 days	RNAseq with hippocampus of six-month-old APP/PS1 mice	Hao and Feng (2021)
				Reduction of Aβ plaques, markedly enhanced mitochondrial autophagy observed in hippocampal neurons	
Male Wistar rat	Streptozotocin (STZ)-induced AD model	i.p. treatment of CBD (20 mg/kg BW)	—	Enhances the brain glucose metabolism	de Paula Faria et al. (2022)
4-month-old TAU58/2 male mice	Genetically modified (tauopathy model)	50 mg/kg CBD i.p. administration	3 weeks	Did not affect behavioral changes	Watt, et al. (2020)
14-month-old TAU58/2 female mice	Genetically modified (tauopathy model)	100 mg/kg CBD i.p. administration	3 weeks	Improving spatial memory along with reducing anxiety-like behaviors and contextual fear-associated freezing	Kreilaus et al. (2022)

To have a mechanistic insight into CBD’s various functions on AD-bearing animals, Hao et al. have used transcriptomic approaches. Authors have performed RNASeq using the hippocampus of six-month-old *APPxPS1* mice treated (i.p.) with 5 mg/kg BW of CBD daily for 30 days. Using bioinformatics-based prediction tools (Gene Set Enrichment Analysis (GSEA) and Kyoto Encyclopedia of Genes and Genomes (KEGG) pathway analysis), authors have reported the reduction of Aβ plaques in CBD-treated mice which is related to improved immune response and enhanced mitochondrial autophagy in hippocampal neurons (Hao and Feng 2021). In another recent study using 5xFAD mice (which mimic early-onset familial AD), Hesam et al. reported that CBD treatment enhances the expression of IL-33 and triggers receptors expressed on myeloid cells 2 (TREM2). IL-33 is a cytokine and TREM2 is a microglial surface receptor, both of which skew microglia in enhancing Aβ clearance, and CBD treatment enhances the expression of such mediators to prevent the cognitive decline in mice (Khodadadi É et al., 2021). More recently, Faria et al. have investigated the effect of CBD on preventing the hypometabolism observed in AD, and have found that 20 mg/kg BW of CBD enhances the brain glucose metabolism in a streptozotocin (STZ)-induced AD model of the male Wistar rat (de Paula Faria et al., 2022).

In addition to the amyloid β model, researchers have also investigated the effect of CBD in the tauopathy model of AD. Recently, Alali et al. have reported that CBD can inhibit aggregation of tau protein (Alali et al., 2021). Using *in vitro* and *in silico* biochemical methods such as circular dichroism (CD) and atomic force microscopy (AFM), the author has demonstrated that CBD molecules bind to recombinant human tau protein and inhibit its aggregation. TAU58/2 transgenic mice, containing a P301S mutation in human tau which emphasizes tau pathogenesis of AD, are used as tauopathy rodent models of AD. Behavioral characteristics for anxiety, cognition, and motor functions have been investigated in the presence and absence of CBD in TAU58/2 transgenic mouse. Although chronic CBD treatment of 50 mg/KgBW for 3 weeks (i.p.) did not affect behavioral changes in such mice (Watt et al., 2020), a higher dose (100 mg/kg) of CBD is reported to be effective in improving spatial memory along with reducing anxiety-like behaviors and contextual fear-associated freezing in all mice (Kreilaus et al., 2022).

CBD has also been reported to combine with THC or other drugs. Sativex, the mixed cannabis (THC + CBD), reduces learning impairment and Aβ-42 peptide levels in AβPP/PS1 transgenic mice when chronically administered during the early symptomatic stage (Aso et al., 2015). Authors have demonstrated that this anti-Alzheimer efficacy of the combined cannabis (THC + CBD) is *via* reduction of astrogliosis, microgliosis, and inflammatory-related molecules in *APPxPS1* mice. Using a genome-wide gene expression study, the author has identified that the redox protein thioredoxin-2 and the signaling protein Wnt16 are the significant targets for the (THC + CBD)-induced effects. The same group has also reported that the combined cannabinoids (THC + CBD) can improve memory impairment in *APPxPS1* mice with an advanced stage of AD, which is not associated with APP processing or reducing glial activity, leading to Aβ deposition as observed in the early stages but *via* reduced GluR2/3 and increased levels of GABA-A Rα1 (Aso et al., 2016). GluR2/3 is a subtype of an AMPA glutamate receptor, and GABA-A Rα1 is a receptor subunit of the GABAergic system which plays a role in preventing neurodegeneration in both AD and in aging. Reduced GluR2/3 can prevent glutamate toxicity, while enhanced GABA-A Rα1 expression plays a role in preventing neuronal dysfunction. Authors have demonstrated that this effect of combined cannabinoids, however, is AD-specific and does not affect the cognitive impairment in healthy aging as observed in wild-type mice. The same group has also investigated if there is any role of the CB2 receptor in this combined anti-AD effect of (THC + CBD) using CB2 knockout *APPxPS1* mice. They have found that although the CB2 receptor has no significant role in the therapeutic benefit of (THC + CBD), the lack of CB2 receptor interferes with the APP processing to reduce the cortical Aβ deposition by enhancing the levels of soluble Aβ40 (Aso et al., 2016).

To further enhance the therapeutic efficacy of CBD, it is chemically conjugated to a fragment of an AChE inhibitor. The new cannabidiol−carbamate hybrid lead compound (namely, C16) exhibits a much stronger inhibition of butyrylcholinesterase (BuChE) (IC_50_ = 5.3 nM) than that of CBD (IC_50_ = 0.67 μM) and crosses the BBB. Authors have demonstrated that C16 is able to improve scopolamine-induced cognition impairment in mice, and the treated mice exhibit better behavioral activity than donepezil (Jiang et al., 2021). Contrary to other observations, Amini et al. have reported that CBD, when delivered using chitosan nanoparticles, reduces Aβ plaque formation and learning and memory in AD-bearing rats *via* enhancing the expression levels of CB1 and CB2, though the effect of empty chitosan NPs on CB1 and CB2 has not been investigated in this study (Amini and Abdolmaleki, 2021).

A clinical trial with THC-free CBD oil has just started (Leszko and Meenrajan, 2021). In Poland, 63 caregivers out of 73 reported CBD to be effective in managing behavioral symptoms of AD, which indicates the initial promise of CBD oil in the clinical study. Collectively, the non-psychoactive CBD holds significant promises in managing behavioral deficit of AD due to its antioxidant and anti-inflammatory properties with a note that its dose and frequency of treatment should be thoroughly investigated in detail in near future.

## Conclusion and future perspectives

Despite the significant advancement in neurology, the cure of neurodegenerative diseases such as AD and PD continues to remain an unmet challenge. Emerging evidence indicates that oxidative stress and inflammation are common amplifiers of disease progression, and the inhibition of neuroinflammation may result in clinical benefit in treating NDs. However, care should be taken when choosing anti-inflammatory drugs that may have a potential adverse effect due to activation of the innate immune system**.** In addition, as neurodegenerative diseases require long-term therapy, the high safety profile of the drug is a crucial factor. Furthermore, to be clinically effective, the therapeutics should gain access to the CNS and specifically target the cells or pathways. To this end, non-psychoactive phytocannabinoid CBD holds significant promises due to their antioxidant and anti-inflammatory properties, high safety profile, and good tolerability. Specifically, neuroprotection *via* PPARγ activation by CBD may hold significant promise as rosiglitazone, an agonist of PPARγ, exhibited prominent clinical benefit either alone (phase 2 clinical trials) or in combination with donepezil (Phase 3) in treating AD patients. However, a thorough investigation of the required dosages and treatment windows in different transgenic mouse models is necessary. Importantly, rodent age and sex are crucial factors when evaluating the therapeutic effectiveness of CBD in some neurodegenerative diseases.

Clinical application of CBD has just started with FDA approval of Epidiolex (CBD in oil formulation) in 2018 for treating pediatric epilepsy. However, despite its multi-faceted therapeutic potential, its clinical approval is impeded by intrinsic characteristics such as poor bioavailability and variable pharmacokinetics profile (Millar et al., 2020). Potential ways to overcome this including usage of drug delivery systems and/or different routes of drug administration are in the preclinical and clinical stages of development. For example, to avoid the precipitation of CBD in the gastrointestinal tract, CBD is used in formulation with oils, surfactants, solvents, or cyclodextrin carriers which are being investigated while the *i.p.* route of administration exhibits a better brain accumulation than the oral route. Considering the general trend of PK study regarding a small percentage of brain accumulation of therapeutics, nano- or micro-sized droplets of such formulations have gained significant importance. In addition, oxidative degradation and drug–drug interaction (when used in combination) of CBD are crucial factors that should be taken care of. To this end, investigation of lipid-based nanocarriers that can solubilize CBD in lipid core has just started (Aparicio-Blanco et al., 2019). Herein, the brain-targeting lipophilic nanocarrier which can solubilize the CBD in its lipid core and deliver a payload to the brain holds significant promise. Collectively, CBD is harmless at a low dose, and preclinical studies indicate its beneficial neuroprotective effect. Clinical trials for CBD in conventional neurodegenerative diseases have started showing its initial promise, especially in improving behavioral health. Thus, CBD may find its clinical application beyond pediatric epilepsy not only to conventional neurodegenerative diseases but also to neurodegenerative conditions secondary to other CNS complications either alone or as an adjuvant in the near future.
